# Biosorption of Uranyl Ions from Aqueous Solution by *Parachlorella* sp. AA1

**DOI:** 10.3390/ijerph18073641

**Published:** 2021-03-31

**Authors:** Ja-Young Yoon, In-Hyun Nam, Min-Ho Yoon

**Affiliations:** 1 Geologic Environment Research Division, Korea Institute of Geoscience and Mineral Resources (KIGAM), Daejeon 34132, Korea; dbswkdud93@naver.com; 2 Department of Bio Environmental Chemistry, Chungnam National University, Daejeon 34134, Korea

**Keywords:** biosorption, uranyl ions, XANES analysis, microalgae, *Parachlorella* sp. AA1

## Abstract

In the present study we investigated the ability of the microalgal strain *Parachlorella* sp. AA1 to biologically uptake a radionuclide waste material. Batch experiments were conducted to investigate the biosorption of uranyl ions (U(VI)) in the 0.5–50.0 mg/L concentration range by strain AA1. The results showed that AA1 biomass could uptake U(VI). The highest removal efficiency and biosorption capacity (95.6%) occurred within 60 h at an initial U(VI) concentration of 20 mg/L. The optimum pH for biosorption was 9.0 at a temperature of 25 °C. X-ray absorption near edge structure analysis confirmed the presence of U(VI) in pellets of *Parachlorella* sp. AA1 cells. The biosorption methods investigated here may be useful in the treatment and disposal of nuclides and heavy metals in diverse wastewaters.

## 1. Introduction

Increased industrial activity has spread environmental contamination and the deterioration of some ecosystems through the accumulation of pollutants including heavy metals, anthropogenic chemicals, and liquids of nuclear waste [1,2]. Mining and metallurgical wastewaters are crucial sources of heavy metal pollution, and the amount of radioactive wastewater discharge is increasing each year with rapid development of the nuclear industry [1,2]. Therefore, the proper treatment and disposal of radioactive wastewater is essential for environmental safety and human health. Biosorption is regarded to be a possible process for the removal of both toxic metals and radionuclides from solution [2,3].

Uranium compounds are diffuse pollutants that comprise one of the threatening heavy metals because of its toxicity and radioactivity [4,5,6]. For uranium contamination, mining (41.14%) and groundwater (39.67%) are the most prominent sources, followed by fertilizer (7.57%), nuclear facilities (7.25%), and the military (4.36%), aside from natural uranium contamination due to the geologic processes [7]. It has been reported that diverse mechanisms through which biotic processing of uranium compounds are usual in the environment, including bioaccumulation, biotransformation, biomineralization, and biosorption [5,6,8,9]. Consequently, microbial communities can also have marked effects on the biosorption of uranium compounds [10,11].

Biological sorption describes the binding of heavy metals to biomolecules or sorption on the surface of cells from an aqueous solution, which subsequently prevents contaminant release [12]. Biological methods including biosorption, bioaccumulation, and bioimmobilization may provide an interesting alternative to heavy metal ion removal methods [4]. Uranium compounds comprise more than 160 mineral species that account for 5% of all known minerals [13]. Once released, the environmental fate and transport of these radionuclides is remarkably controlled by microbial activity, as natural microbial flora have diverse mechanisms for interacting with such metallic pollutants, including reductive precipitation, solubilization, and biosorption/bioaccumulation; these processes finally determine the environmental toxicity and mobility of metallic pollutants [5]. Microalgal uptake of nuclides has received much recent attention among radioactive wastewater treatment technologies, and selection of the most effective microalgal species for uranyl ions biosorption is a major focus for the research and applications in this area [12].

The aim of the present study was to investigate the ability of *Parachlorella* sp. AA1 of green microalga to grow in the presence of uranyl ions ((UO_2_)^2+^, U(VI)), and to biosorb it. *Parachlorella* sp. AA1 was cultivated in the presence of various concentrations of U(VI) to assess the physiological effects of U(VI) on the microalga, and to investigate its biosorption capacity. Inductively coupled plasma mass spectrometry was used to determine the amount of U(VI) that accumulated in the biomass, and X-ray absorption near edge structure spectroscopy was used for verification of the extent of accumulation and to investigate U(VI) speciation. This work describes the potential use of microalgae as a promising new biological alternative to existing technologies for the biosorption of U(VI) with the advantages of high efficiency, applicability, and economic feasibility, and for the removal and recovery of radionuclides such as uranyl ions from low level wastes.

## 2. Materials and Methods

### 2.1. Strain and Culture Conditions

The unicellular non-motile green microalga *Parachlorella* sp. AA1 was obtained from the University of KwaZulu-Natal (Pietermaritzburg, South Africa) in solid modified BG11 medium (https://biocyclopedia.com/index/algae/algal_culturing/bg11_medium_composition.php) (accessed on 16 March 2021). The BG11 medium was prepared using distilled water and the purified cells were suspended in 5 mL sterile BG11 medium. A stock solution of AA1 was cultivated in liquid BG11 medium in Erlenmeyer flasks. The flasks were incubated at 25 °C with continuous aeration at a light intensity of 50 W/m^2^ (60 W halogen lamp) light on a 12 h/12 h light/dark cycle. Cell cultures of AA1 were propagated in BG11 medium in Erlenmeyer flasks, and were subcultured by serial transfer to fresh medium every 5 days to ensure the cells were in late exponential growth phase; the cultures were regularly assessed to prevent contamination. The morphological features of AA1 were observed and photographed using light microscopy (DM-500, Leica, Germany).

### 2.2. Preparation of U(VI) Solutions

A stock solution of U(VI) (1000 mg/L) was made by dissolving uranyl acetate dihydrate UO_2_(OCOCH_3_)_2_·2H_2_O in a small amount of concentrated nitric acid, and diluting to 1000 mL. The UO_2_(OCOCH_3_)_2_·2H_2_O was obtained from Accustandard (New Haven, CT, USA). Working solutions from 0.5 mg/L to 50.0 mg/L concentrations of U(VI) were prepared from the above U(VI) stock solution. All solutions were prepared using distilled water. Other chemical reagents used in the experiments were of the highest grade that was commercially available.

### 2.3. Determination of Growth Conditions

To investigate its optimal growth conditions, cells of AA1 were cultivated under aerobic conditions in BG11 medium at pH values ranging from 5.0 to 9.0. To determine the optimal initial pH and U(VI) concentration conditions, the culture media were prepared as described above, and the initial pH of the U(VI) solution was 7.2 and adjusted with 0.10 mol/L HCl or NaOH. The temperature of growth and pH were 25.0 °C and 9.0 ± 0.02, respectively, and the light conditions were as described above. To ensure aerobic conditions the cultures were agitated on an orbital incubating shaker (Daehan Sci., Wonju, Korea) with 150 rpm rotation speed. The AA1 cells were cultivated in 500 mL Erlenmeyer flasks with a working volume of 200 mL. The growth of AA1 was monitored by counting the cells in 10–20 μL volumes of cell suspension in a hemocytometer counting chamber (V-slash Neubauer-improved Marienfeld 0650030, using a 0.4 mm cover glass, Germany) using light microscopy (DM-500, Leica, Germany). For confirmation of the growth rates, cell growth was also monitored by optical density (OD_680_) measurements using a UV–Vis spectrophotometer (Biochrom, Cambridge, UK). The growth rate was determined as described previously [14] and calculated as: growth rate = (ln OD_t_ − ln OD_0_)/t, where OD_0_ is the initial value of OD_680_ and OD_t_ is the value of OD_680_ after t days.

### 2.4. U(VI) Biosorption Experiments

To investigate U(VI) biosorption, AA1 cells in the exponential growth phase were collected by centrifugation at 6000 rpm for 15 min. The cells were washed three times in sterile 20 mM phosphate buffer and resuspended in 10 mL of the same buffer. A known volume of each cell suspension (equivalent to 1 mg dry mass per ml of U(VI) solution) was added, and the solution was mixed and incubated at 25 °C with shaking (150 rpm) for 12 h in a growth chamber at a light intensity of 50 W/m^2^ (halogen lamp; 60 W). After each biosorption experiment was completed, the biomass was separated by centrifugation at 6000 rpm for 15 min, and the U(VI) content of the supernatant and pellet was assessed using a HPLC-ICP-MS (Agilent 7800, Santa Clara, CA, USA). The separated supernatant was filtered using a 0.1 μm syringe filter and diluted with 2% nitric acid solution. Cell pellets were suspended in 50 µL of nitric acid and left to digest for 4 h for U(VI) analysis using HPLC-ICP-MS. The volume of each supernatant or pellet sample was reduced to approximately 30–40 µL after digestion, then 1 mL of 1% (*v*/*v*) nitric acid diluent was added to each cell sample. Measurements were performed using an HPLC-ICP-MS instrument under operating conditions suitable for usual multi-element analysis. The instrument calibration was performed using 0, 2, 5, 10, 20, 50, 100, and 500 ppb certified ICP-MS standards (Accustandard, New Haven, CT, USA) for a range of elements prepared in 1% (*v*/*v*) nitric acid.

### 2.5. X-ray Absorption near Edge Structure Analysis

All samples for analysis using X-ray absorption near edge structure (XANES) spectroscopy were prepared following U(VI) biosorption experiments, using AA1 cell pellets after centrifugation and overnight freeze drying. XANES U spectra were obtained at the Pohang Accelerator Laboratory (Pohang, Korea) using beamline 8C. The L3-edge of the U XANES spectra were collected in fluorescence mode using a SDD detector in the He-flow chamber to avoid oxygen contact during the measurement. Samples were mounted on metal plate, sealed with Kapton tape, and oriented at 45° to the incident X-ray beam. Samples were ground, and packed in a 2 mm depth aluminum holder. The reference was 9 mL of 1000 ppm U(VI) standard solution, which was added in 3 g SiO_2_ powder and freeze-dried for comparison with U(VI)-biosorbed AA1 cells.

## 3. Results and Discussion

### 3.1. AA1 Strain Properties

**Figure 1:**
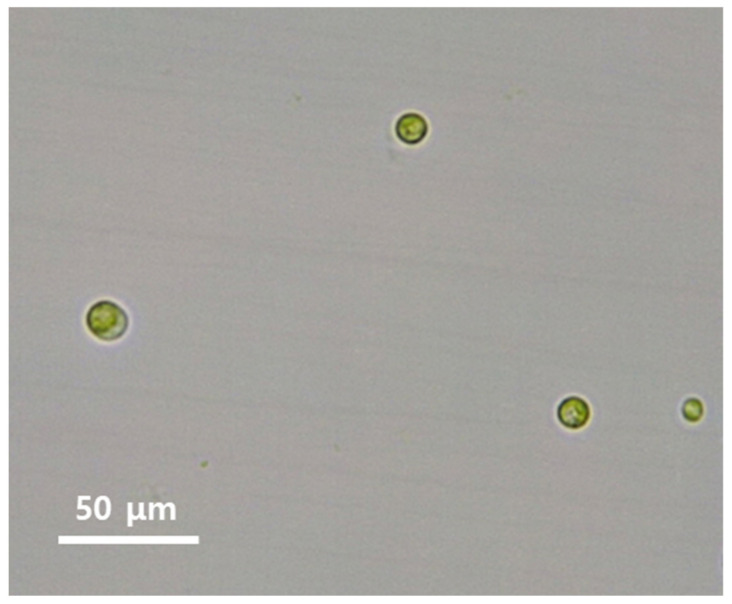
Morphology of *Parachlorella* sp. AA1 in U(VI) biosorption experiments. The shape of the strain AA1 cells was not disturbed after contact with the U(VI) containing solution.

By light microscopy the microalgal strain *Parachlorella* sp. AA1 was unicellular, yellow-green, spherical, and 5–15 μm in diameter (Figure 1). The chloroplast contained a large pyrenoid, and was single, irregular laminate, parietal, and occupied most of the space in the cell. Small globules that refracted light were also evident in the cells. In more mature cells, two or four autospores formed in the mother cell. After the spores were released, some of the resulting cells formed into a mucilaginous mass (palmella). Based on its morphological and reproductive characteristics, the strain was assigned to the genus *Chlorella* (Chlorophyta), as previously reported [15,16]. A small subunit ribosomal RNA gene sequence of AA1 was deposited in the NCBI (National Center for Biotechnology Information) as MT984303. The genus *Parachlorella* was established by Krienitz et al. (2004) [17], based on 18S rDNA and ITS sequences. It is a unicellular planktonic eukaryote that comprises solitary cells or cells gathered in groups. In addition, it could be observed that the morphology of *Parachlorella* sp. AA1 cell was maintained after contact with U(VI), as shown in Figure 1.

### 3.2. Effects of Initial pH and U(VI) Concentration

Solution pH is a critical parameter that influences U(VI) biosorption. The U(VI) in solution and the surface charge on microorganisms are both pH dependent [18]. Experiments on the biosorption of U(VI) at pH values ranging from 5.0 to 9.0 (Table 1) showed that cell growth and U(VI) biosorption rates were greatest at pH 9.0, but decreased over the range pH 5.0–8.0. At pH 9.0 the maximum optical density was 0.548 ± 0.015 and the maximum U(VI) biosorption rate was 97.2 ± 0.43%.

The uptake of U(VI) is a complicated process that is reliance on the element chemistry and the cell properties of the microalgal strain involved. Among the critical factors controlling the uptake of U(VI) were the pH conditions, which determined the U species available in solution and influenced the surface charge on the AA1 cells, and the U(VI) concentration. At low pH values, cationic U species are generally dominant [19]. It was noted that the biosorption process was dependent on the solution initial pH value. The biosorbed metal ion extents increased with increasing solution pH, and the maximum removal efficiency (~97.2%) was observed at pH 9.0. At acidic pH values, the uptake of metal ions was inhibited, which can be influenced to the being of H_3_O^+^ ions competing with the U species for contact sites [20]. Under low pH conditions, the U is present in solution mostly in the form of free UO_2_^2+^ ions, which compete with protons for adsorption sites on the algal biomass, whereas at higher pH values the formation of hydroxy-uranyl species occurs. As shown in Table 1, the biosorption of U(VI) was maximum at pH 9.0 (97.2%), and decreased to 62.6% at pH 5.0. Under high-pH conditions, the speciation of U is dominant due to a series of aqueous carbonate complexes, which increase the U solubility like these environmental conditions. Thus, pH 9.0 was found to enable maximum biosorption, and was employed in all subsequent experiments. Previous comparable results have been reported elsewhere for U(VI) biosorption [21,22,23].

The results of experiments assessing the effect of initial U(VI) concentration (0.5–50 mg/L) on biosorption are shown in Table 2.

For initial medium concentrations of U(VI) ranging from 0.5 to 50.0 mg/L, the maximum U(VI) removal efficiency (97.4%) occurred at a concentration of 20 mg/L. The U(VI) biosorption capacity of AA1 increased with increasing U(VI) concentration from 0.5 to 20.0 mg U(VI)/L, but decreased when the concentration exceeded 20.0 mg/L, indicating that AA1 reached saturation at 20 mg/L, and that U(VI) biosorption capacity did not increase when the saturation concentration of 20.0 mg/L was exceeded. As the initial concentrations of U(VI) increased, the cell density could not increase; therefore, it was estimated that the adsorption rates decreased after saturation point. Therefore, the mechanism of U(VI) biosorption process could be considered as the adsorption to the cell surface. This is probably related to the fact that the number of active biosorption sites is determined by the concentration of AA1 cells, whereby high solution U(VI) concentrations can saturate the active sites and prevent further biosorption. Past studies have described that the collision frequency between metal ions and the adsorbent materials increased with increasing initial metal ion concentration, causing enhanced adsorption processes [24].

**Figure 2:**
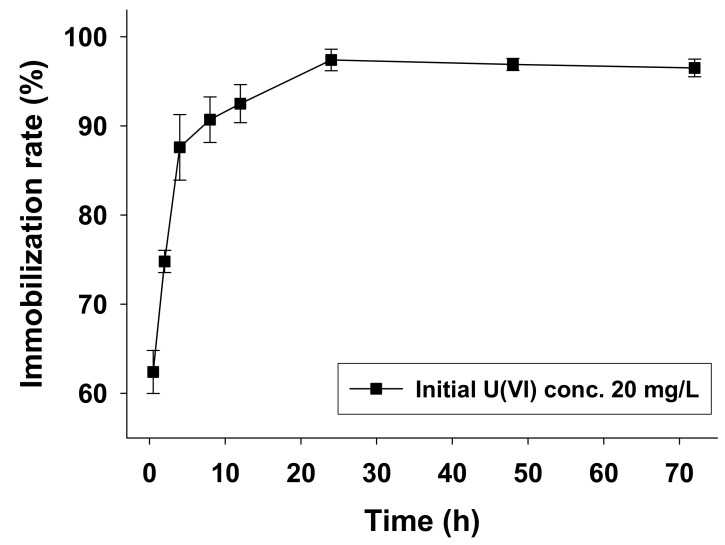
The effect of time on the uptake of U(VI) by *Parachlorella* sp. AA1 (C_init_ 20 mg/L, pH_init_ 9.0).

Figure 2 shows the effect of contact times up to 72 h on biosorption of U(VI) by AA1 cells with an initial medium U(VI) concentration of 20 mg/L at pH 9.0 and 25 °C.

The biosorption rate increased rapidly from 0.5 to 4 h, increased slightly from 4 to 24 h, and stabilized thereafter. This pattern of biological adsorption for metal ions has been reported previously [2,22,25]. Taken together, the optimal conditions for U(VI) biosorption by *Parachlorella* sp. AA1 were pH 9.0, a temperature of 25 °C, an initial U(VI) concentration of 20 mg/L, and a contact time of 24 h.

### 3.3. U(VI) Biosorption Experiments

**Figure 3:**
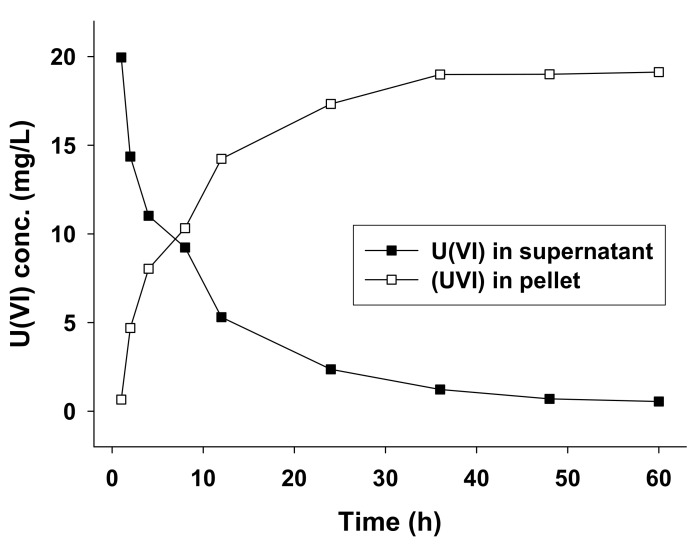
Biosorption of U(VI) from culture suspension by *Parachlorella* sp. AA1, evidenced by depletion of U(VI) in the supernatant and U(VI) accumulation in the algal cells. The supernatant and *Parachlorella* sp. AA1 cells were separated by centrifugation, and the U(VI) concentrations in each were measured using LC-ICP-MS. The batch experiments involved three independent replicates.

Experiments investigating U(VI) biosorption by AA1 cultures were conducted under optimum conditions (pH 9.0, temperature 25 °C) for 60 h. Pre-grown cells of AA1 were inoculated into a series of 500 mL Erlenmeyer flasks containing 200 mL of culture suspension containing the initial U(VI) concentration (20 mg/L). The number of AA1 cells in the culture suspension was approximately 2.0 × 10^7^ (determined using a hemocytometer). Figure 3 shows that there was a 97.5% reduction in the concentration of U(VI) in the supernatant over 60 h of incubation, and a corresponding increase in the U(VI) concentration in the pellet over the same time period; the U(VI) biosorbed in the pellet represented 95.6% of the initial concentration over the 60 h of the experiment. After 36 h of incubation, the concentrations of U(VI) in the pellet and supernatant stabilized, consistent with saturation of biosorption sites in AA1. Further field application studies may improve our understanding of microalgal U(VI) biosorption in the environment and help optimize our efforts to remediate U(VI) contaminated wastewaters.

Previous studies have investigated the ability of many species (including sea vegetables, microalgae, fungi, bacteria and yeasts) to sequester heavy metals from dilute aqueous solutions [22,23,26,27,28,29]. The removal of uranium compounds from aqueous media can occur through various processes, including complexation, ion exchange, membrane separation, chemical precipitation, adsorption using both synthetic and natural sorbents and biological sorption [20,30,31,32,33]. Biosorption has received significant focus, including our study, and is regarded as an innovative technology with the potential to replace common processes for remediating metal contamination in wastewaters. It provides advantages including high sorption capacity, low operating cost, high efficiency in detoxification of dilute effluents, easy regeneration, volume reduction of disposable sludge, and high environmental sustainability [25,34,35]. Therefore, the results of the biosorption experiments show that *Parachlorella* sp. AA1 may be applicable to treatment of U(VI)-containing wastewaters because the technique is relatively simple, rapid, easy to apply, and shows high removal rates. Our study provides novel observations regarding the uptake of U(VI) by biomass of the microalga *Parachlorella* sp., and its ability to biosorb this radionuclide.

### 3.4. Detection of Biosorption of U(VI) Using XANES Analysis

**Figure 4:**
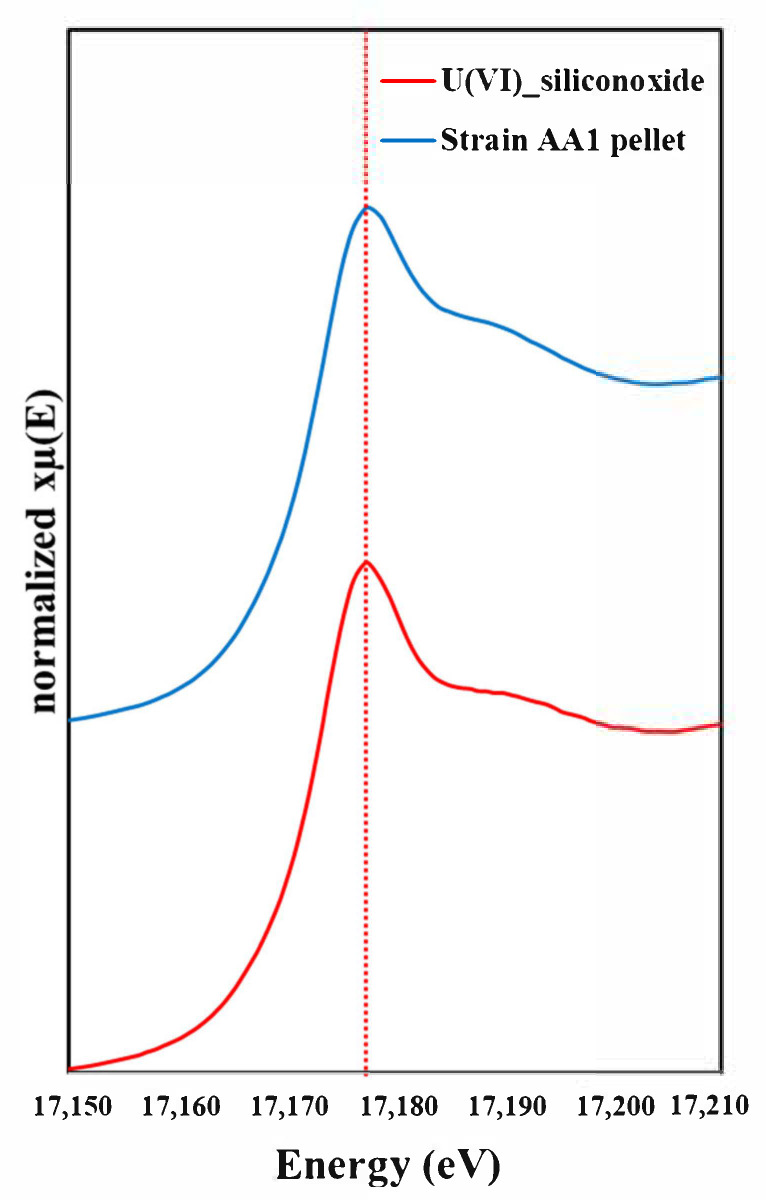
L3-edge XANES spectra of U(VI) biosorbed by AA1, measured using a He flow chamber to maintain anaerobic conditions. The blue line shows the spectrum for a freeze-dried sample of the U(VI) biosorbed on *Parachlorella* sp. AA1 cells, and the red line shows the spectrum for a U(VI) reference standard solution. The red dotted line shows the U(VI) peak.

To confirm the biosorption of U(VI) in pellets of *Parachlorella* sp. AA1 we used XANES analysis (Figure 4).

U L3-edge XANES spectra of *Parachlorella* sp. AA1 pellet obtained following biosorption experiments using 20 mg/L of U(VI) using a He flow chamber to keep anaerobic condition. In this figure, red dotted line marks the location of U(VI) peak. The results show that the experimental U(VI) and the authentic U(VI) standard were both biosorbed by the algal cells. Collectively, our results demonstrate that *Parachlorella* sp. AA1 could potentially be used in the biological treatment of U(VI) from radionuclide-containing wastewater.

## 4. Conclusions

The biosorption of U(VI) by *Parachlorella* sp. AA1 was studied using a batch technique under various experimental conditions. The maximum biosorption efficiency was found to be 95.6% within 60 h under the optimized experimental conditions (pH 9.0; C_i_ = 20 mg U(VI)/L; 25 °C). Rapid biosorption on *Parachlorella* sp. AA1 cells in the initial stages of equilibration was investigated, and the phenomenon was measured using LC-ICP-MS and XANES analyses. The results of this study show that the microalga *Parachlorella* sp. AA1 can effectively remove U(VI) from aqueous solution, and is potentially a suitable biosorbent treatment that is non-toxic, low cost, biodegradable, environmentally friendly, and biocompatible. Further enzymatic and genetic studies will expand our understanding of the process of U(VI) biosorption on this microalgal species, and progress efforts to remediate radioactive wastewaters.

## Figures and Tables

**Table 1:** The effect of medium pH on the growth of AA1 cells and their rate of U(VI) biosorption ^a^.

Initial pH of Medium	Optical Density (680 nm) ^b^	Biosorption Rate (mean ± SD ^b^ %) ^c^
5.0	0.297 ± 0.007	62.6 ± 1.99
6.0	0.385 ± 0.010	71.5 ± 1.12
7.0	0.501 ± 0.009	82.2 ± 0.84
8.0	0.535 ± 0.011	90.5 ± 0.75
9.0	0.548 ± 0.015	97.2 ± 0.43

^a^ The U(VI) biosorption rate was determined after 24 h incubation. ^b^ Means and standard deviations were obtained from three independent replicates. ^c^ All values were determined in triplicate (*p* = 0.001).

**Table 2:** Effect of initial U(VI) concentration on biosorption by *Parachlorella* sp. AA1 cells ^a^.

Initial U(VI) Concentration (mg/L)	Biosorption Rate (mean ± SD ^b^ %) ^c^
0.5	95.7 ± 0.59
1.0	96.2 ± 1.03
5.0	96.8 ± 0.44
10.0	96.9 ± 0.81
20.0	97.4 ± 0.34
30.0	91.2 ± 0.16
50.0	89.8 ± 0.93

^a^ The U(VI) in the supernatant was determined after 24 h incubation. ^b^ The means and standard deviations were obtained from three independent replicates. ^c^ All values were determined in triplicate (*p* = 0.001).

## Data Availability

The data that support the findings of this study are available from the corresponding author, upon reasonable request.
